# A closer look at the role of nutrition in children and adults with ADHD and neurodivergence

**DOI:** 10.3389/fnut.2025.1586925

**Published:** 2025-07-30

**Authors:** Catherine Hunter, Carla Smith, Emily Davies, Simon C. Dyall, Rachel V. Gow

**Affiliations:** ^1^School of Life and Health Sciences, University of Roehampton, London, United Kingdom; ^2^Independent Researcher, Nutritious Minds Trust Charity, Surrey, United Kingdom; ^3^Department of Psychology, St Mary’s University, London, United Kingdom

**Keywords:** ADHD, Autism Spectrum Disorders, brain health, brain-selective-nutrients, omega-3-fatty acid, neurodivergence, nutrition, nutritional psychiatry

## Abstract

**Introduction:**

The role of nutrition in Attention-Deficit, Hyperactivity Disorder (ADHD) and other neurodivergent conditions is of growing public and research interest. There is little research reporting vitamin, mineral and omega-3 fatty acid levels in ADHD and brain health.

**Methods:**

This study presents nutritional and psychological data from a community UK sample of children (*n* = 47, *Mean age*: 10.1 years) and adults (*n* = 10, *Mean age*: 29.8 years) with ADHD, autism, dyslexia and other neurodivergent conditions (total *n* = 57). The participants undertook a blood draw which measured a range of vitamins, minerals and omega-3 fatty acids as well as food allergies and food intolerances which were then correlated with psychological symptom scores measuring ADHD symptoms.

**Results:**

The key findings, revealed that both children and adults presented with a range of insufficiencies in key nutrients which facilitate neurotransmitter function and, which are deemed as brain-essential, namely omega-3 fatty acids, zinc, B-vitamins and vitamin D. Furthermore, significant relationships were observed between nutrient levels and ADHD symptom severity in the children’s group. For example, red blood cell magnesium was negatively correlated with the Conners CI-Parent Rating Scale (CPRS) Disruptive Behavior scores (rho = −0.597, *p* = 0.024). The omega-3 index (sum of EPA + DHA as a percentage of total fatty acids) was negatively correlated with their Learning and Language Disorder scores, (rho = −0.601, *p* = 0.018). Magnesium levels were also associated with overall ADHD symptom severity (rho = −0.612, *p* = 0.02), implying that the greater the severity of ADHD symptoms, the lower the magnesium. This clinical cohort also presented with a range of food intolerances with over 80% of participants presenting with high reactivity scores to cow’s milk, other dairy, and casein, and just over half the sample intolerant to wheat and wheat gluten.

**Discussion:**

This is a novel study which presents preliminary data and insights in the role of nutrition in ADHD and neurodivergence. and relationships between nutritional insufficiencies and ADHD-symptoms. It specifically demonstrates a range of food intolerances and relationships between nutritional insufficiencies and ADHD-symptoms, which warrant further exploration in larger case-control groups.

## 1 Introduction

The role of nutrition in brain health and in particular in Attention-Deficit/Hyperactivity Disorder (ADHD) and other neurodivergent conditions is of growing public and research interest. Neurodivergence is a term employed to describe a range of differences in individual brain functioning, processing and traits that differ from what is considered typical and includes conditions such as ADHD and Autism Spectrum Disorders (ASD). Neurodiversity is a term coined by Judy Singer in the late 1990s and is an umbrella term which can be useful to describe people with a varying behaviors and characteristics of neurodevelopmental conditions in a non-prejudiced way and embraces the notion that different people have different brains and no-one brain is exactly the same as another (1).

Nutritional science has traditionally focused on isolating single nutrients to investigate their effects producing varied and often inconclusive findings. However, it is increasingly recognized that there is no single nutrient responsible for brain health and nutrients interact synergistically to facilitate absorption and yield physiological impacts in the body and brain (2). The Dietary Reference Intakes (DRIs) provide guidelines for nutrient intake and recommendations to prevent nutrient deficiencies. DRIs include four categories of values including recommended dietary allowance (RDA) which is the amount of a nutrient which is adequate to meet the requirements of 97–98% of a population (3). The field of Nutritional Psychiatry is concerned with the relationship between food and the health of the human brain. Furthermore, specifically how “brain-selective” nutrients impact brain activity, structure and function and in turn help regulate mood, behavior, learning and cognition (4).

ADHD is a neurobiological condition with complex etiology influenced by a combination of genetic and environmental factors (5). Symptoms of ADHD present differently in childhood and adulthood and can be broadly characterized by impairing levels of inattentiveness, disorganization, and/or impulsivity/hyperactivity (6). ADHD is considered one of the most common neurodevelopmental conditions impacting around 6–11% of children and 2–6% of adults worldwide (7, 8). ADHD often co-occurs with conditions and/or symptoms of depression, anxiety, dyslexia, dyspraxia, dyscalculia, generalized anxiety disorder (GAD), ASD, obsessive compulsive disorder (OCD), sensory disorders and oppositional defiant (ODD) disorders (9). Diagnostic criteria is outlined in the Diagnostic and Statistical Manual for Mental Disorders Volume 5 (DSM-5) and the International Classification of Diseases 11th Revision (ICD-11) (10). The first line of treatment recommended by National Institute for Health and Care Excellence (NICE) guidelines is behavioural therapies and pharmacological treatment^1^. The number of adults receiving NHS prescriptions for ADHD medication such as methylphenidate (MPH) was reported to have increased seven-fold over the past decade resulting in around 232,000 prescriptions in 2023 (11). However, the long-term effectiveness and safety of stimulant medications remain under investigation. In 2024, a study by Zhang and colleagues reported that ADHD medications can increase risk of cardiovascular disease (each 1-year increase of ADHD medication use was associated with a 4% increased risk of CVD) advising that the potential risks and benefits of long-term ADHD medication use should be carefully considered (12). Meanwhile, there is growing scientific and public interest in the role of nutrition in ADHD and brain activity (13).

The human brain has a specific composition which requires a particular nutrient intake for structure, activity and function (14, 15). The brain is approximately 60% lipid by dry weight (16), and is enriched in long-chain omega-3 polyunsaturated fatty acids (PUFAs), where docosahexaenoic acid (DHA, 22:6n-3) comprises 20–25% of the fatty acid content of neuronal membranes (17, 18), whereas microglia content is higher in eicosapentaenoic acid (EPA, 20:5n-3) than DHA (19). EPA and DHA are also critical for a range of brain functions including cell-signaling, gene expression, myelination, serotoninergic and dopaminergic functioning (20). Inadequate dietary intake of EPA and DHA is linked to a wide-range of psychiatric and neurodevelopmental outcomes including major depressive disorder, anxiety, schizophrenia, psychosis, ASD and ADHD (21–25). The brain also has a requirement for a daily intake of a range of nutrients including B–vitamins, vitamins C and D magnesium, zinc, iron, iodine, and choline, to function optimally (4, 26–28).

Neumann et al. (29) recently identified that lower methylation status at birth was associated with later development of ADHD symptoms. These findings report that DNA methylation may exert an influence on ADHD symptoms, potentially via modification of neurotransmitter functioning or a process called neurite outgrowth which has implications for the field of Nutritional Psychiatry (29). Nutrition plays a critical role in DNA methylation and, in particular, omega-3 fatty acids have been found to decrease DNA methylation and restore neurite outgrowth (30–32). Omega-3 fatty acids have anti-inflammatory and pro-resolving properties and are known to modify gene expression within cells (33). A study by Karimi et al. (34)—and there are other studies not reviewed here—reported that DHA–rich omega-3 fatty acid supplementation decreases DNA methylation (34–36). The omega-3 index is a measure of the sum of EPA and DHA in red blood cells and is increasingly being applied to neuropsychological conditions including ADHD and autism [*Neuroimaging, Omega-3 and Reward in Adults With ADHD (NORAA) Trial*, 2014; 37)]. The adult omega-3 index presents a range from optimal at 8–12%, intermediate: 4–8% and suboptimal at 0–4%, and it has been suggested that everyone should be in the optimal range for human health (38). Other nutrients which may play a role in lowering risk of DNA methylation include folate, flavonoids, choline, resveratrol, sulforaphane, curcumin and B-vitamins (39, 40).

Individuals with neurodivergent conditions such as ADHD and ASD are known to present with problematic food relationships including Avoidant Restrictive Food Intake Disorder (ARFID). The exact reason for this remains unclear but is likely to be multifactorial and mediated by selective eating, sensory issues, and food avoidance. Emerging research has implicated the role of gut microbiome in ADHD and ASD and increased risk of gut dysbiosis which can act as a catalyst for poor mental health (41–43). Diet plays a key role in modulating gut microbiome which in turn impacts the gut-brain axis (44, 45). Nutritional supplementation studies have provided insights into the influence of specific nutrients such as pre- and probiotics in modulating both stress, immune and neuronal function (46–48). However, food sensitivities in ASD and ADHD are arguably underexplored and often limited to case-control findings. For example, food intolerance and food allergy data are confined to functional medicine and nutrition clinics. There is increasing awareness, but often in isolated pockets of research, of the emerging connection between food cravings, gastrointestinal (GI) issues, selective eating habits, food sensitivities, and gut health. These complex interactions between dietary-related behaviors and food intake, nutritional insufficiencies, gut health, and neurodevelopment are an expanding field of study warranting closer exploration.

Lower levels of specific nutrients including iodine, folate, B-vitamins, iron, zinc and omega-3s have been observed in children with ADHD (49–52). Meanwhile, supplementation with omega-3, B-vitamins, zinc and magnesium has been independently found in clinical trials to improve ADHD symptoms (52–56). For example, it is well established that vitamin B6 (pyridoxine) serves as a coenzyme in numerous enzymatic processes and furthermore supports the synthesis of neurotransmitters—dopamine, serotonin, gamma-aminobutyric acid (GABA) and norepinephrine—helping to maintain a balance critical for cognitive and emotional regulation (57–59). Furthermore, dysregulation of these neurotransmitters is considered to be a hallmark feature of ADHD.

This preliminary study aimed to establish nutrient profiles of children and adults in the community with ADHD, ASD and other neurodivergent symptoms. This study presents biochemical findings from nutritional data which examined key nutrients linked to neurotransmitter function (e.g., omega-3 fatty acids, zinc, magnesium, iron, iodine, Vitamin D, B–vitamins). The study sought to specifically examine nutritional insufficiencies employing Dietary Reference Intakes (DRIs) as well as food intolerances and their relationships to ADHD symptoms.

### 1.1 Study aims

This study aimed to assess whether blood levels of key (“brain-selective”) nutrients were lower (i.e., insufficient) than recommended dietary reference intakes in individuals presenting with ADHD and/or similar neurodivergent conditions. Secondary analysis explored relationships between red blood cell (RBC) nutrient levels and ADHD symptom scores as measured by the Conners Parent ADHD Rating Scales (CPRS-RS). The CPRS measures scores from individual subscale scores and included measures of: *Disruptive Behavior Disorder Indicator*, *Learning & Language Disorder Indicator*, *Mood Disorder Indicator*, *Anxiety Disorder Indicator*, and *ADHD Indicator*.

## 2 Materials and methods

The data was previously collected through the private clinic of Dr. Rachel Gow in the context of nutritional and psychological assessments between 2017 and 2024. Participants were screened via structured clinical interviews, standardized psychological assessment, and laboratory analyses of nutritional profiles (see Tables 1, 2).

**TABLE 1:** Demographic data, including diagnoses and symptoms in children and adults with ADHD and other neurodivergent symptoms.

Demographics: diagnoses & symptoms (full cohort, *n* = 57)	Count or *M*	Percentage (%)
**Gender**		
Male	35	61%
Female	22	39%
Mean age	13.5	
**ADHD subtype according to the ChIPS**		
Predominantly inattentive	9	16%
Predominantly hyperactive/impulsive	0	0%
Combined type	11	19%
**Pre-existing ADHD diagnosis**		
Predominantly inattentive	9	16%
Predominantly hyperactive/impulsive	0	0%
Combined	17	30%
**Type of ADHD (CAADID)**		
Predominantly inattentive	0	0%
Hyperactive/impulsive	1	2%
Combined	1	2%
**Any ADHD**	48	84%
**Other diagnosis**		
Any other diagnosis	41	72%
Autism spectrum disorder (ASD)	5	9%
ASD traits	7	16%
Dyspraxia	5	9%
Dyslexia	7	12%
Dyscalcula	1	2%
Dysgraphia	2	4%
Obsessive compulsive disorder (OCD)	2	4%
Epilepsy	1	2%
Depression	7	12%
General anxiety disorder (GAD)	16	28%
Eating disorders	2	4%
Self-harm	3	5%
Hypermobility	4	7%
Tourette’s syndrome	5	9%
Oppositional defiant disorder (ODD)	4	7%
**Parent-reported aggression**		
Trichotillomania	1	2%
Suicide ideation	4	7%
School phobia	3	5%
Speech language difficulties	11	19%
Sensory or auditory processing disorder	8	18%%
Global developmental delay	1	4%

**TABLE 2:** Demographic data including medication, supplements, eating styles, food intolerances, childhood infections, antibiotic use and IQ in children and adults with ADHD and other neurodivergent conditions/symptoms.

ADHD medication	Count or *M*	Percentage (%)
Methylphenidate (Concerta, Ritalin, Focalin)	14	25%
Amphetamines (Adderall, Vyvanse, Dexedrine)	2	4%
Non stimulants (Atomoxetine, Strattera, Clonidine)	1	2%
**Supplements**		
Any supplement	32	56%
Omega-3	19	33%
Multi-vitamin	9	16%
Vitamin D	6	11%
Magnesium	9	16%
Probiotics	2	4%
Vitamin C	4	7%
Melatonin	5	9%
Other supplements (not listed)	8	14%
**Fussy eater, parent-report (*n* = 44)**		
Yes	17	39%
No	27	61%
**Food (IgG) intolerances (*n* = 38)**		
Any intolerance	38	100%
Cow’s milk	32	84%
Egg yolk	18	47%
Egg white	23	61%
Casein	28	74%
Other dairy	33	87%
Wheat	20	53%
Gluten	16	42%
Other cereals and seeds	23	61%
Yeast	11	29%
Candida albicans	16	42%
Nuts	14	37%
Meats	8	21%
Fish	4	11%
Fruits	11	29%
Vegetables	12	32%
**Complications at birth (*n* = 49)**		
C-section	18	33%
Premature birth	1	2%
Umbilical cord around neck	4	8%
**Childhood Infections (*n* = 46)**		
Less than 3	26	57%
3–5	13	28%
5 or more	1	2%
**Antibiotics courses (*n* = 49)**		
1 to 5	20	35%
5 or more	11	19%
**IQ (Kaufman Brief Intelligence Test, Second Edition (KBIT-2)**		
Verbal	110.8±3.3	
Nonverbal	109.1±6.0	
Composite	114.2±3.1	
**Other IQ test scores**		
Verbal	113.4±10.5	
Nonverbal	100.0±8.4	
Composite	101.3±8.9	

### 2.1 Participant & consent

Each family was briefed on the requirements of the project and invited to sign written informed consent. Ethical permission for the project was granted by the University of Roehampton Ethics Committee, reference: LSC 24-400. All data was anonymized, and each participant was allocated a unique (unidentifiable) ID code.

### 2.2 OmegaQuant analytics

A drop of non-fasted whole-blood was collected on filter paper that was pre-treated with a cocktail solution (Fatty Acid Preservative Solution, FAPSTM) and allowed to dry at room temperature for 15 min. The dried blood spots (DBS) were shipped to OmegaQuant for commercial fatty acid analysis. One punch of the DBS was transferred to a screw-cap glass vial followed by addition of methanol containing 14% boron trifluoride, toluene, methanol (35:30:35 v/v/v) (Sigma-Aldrich, St. Louis, MO). The vial was briefly vortexed and heated in a hot bath at 100°C for 45 min. After cooling, hexane (EMD Chemicals, United States) and HPLC grade water was added, the tubes were recapped, vortexed and centrifuged help to separate layers. An aliquot of the hexane layer was transferred to a GC vial. GC was carried out using a GC-2010 Gas Chromatograph (Shimadzu Corporation, Columbia, MD) equipped with a SP-*2560, 100-m fused silica capillary column (0.25 mm internal diameter, 0.2 um film thickness; Supelco, Bellefonte, PA). Fatty acids were identified by comparison with a standard mixture of fatty acids characteristic of red blood cells (GLC OQ-A, NuCheck Prep, Elysian, MN) which was also used to construct individual fatty acid calibration curves. The following 24 fatty acids (by class) were identified: saturated (14:0, 16:0, 18:0, 20:0, 22:0 24:0); cis monounsaturated (16:1, 18:1, 20:1, 24:1); trans (16:1, 18:1*, 18:2); cis n-6 polyunsaturated (18:2, 18:3, 20:2, 20:3, 20:4, 22:4, 22:5); cis n-3 polyunsaturated (18:3, 20:5, 22:5, 22:6). Fatty acid composition was expressed as a percent of total identified fatty acids. The omega-3 index is defined as the sum of EPA and DHA as a percentage of the total measured fatty acids and adjusted by a regression equation (*r* = 0.97) to predict the Omega-3 Index in the RBC (60).

### 2.3 Nutritional blood analysis

The nutritional analysis was conducted either by Biolab (The Stone House, 9 Weymouth Street, London, W1W 6DB) or Viva Health Laboratories (VHL), New Lodge, Drift Rd, Windsor SL4 4RR.

Nutritional data was available for 67% of the participants (*n* = 38). Fasted blood draws were taken by a qualified phlebotomist either at The Hale Clinic, 4 Harley St, London W1G 9PB or Biolab, The Stone House, 9 Weymouth Street, London, W1W 6DB or a home visit. The nutritional profiling included measurements of iron, magnesium, zinc, iodine, vitamin E (as alpha-tocopherol), alpha-carotene, beta-carotene, vitamin B1 (thiamine), vitamin B2 (riboflavin), vitamin B6 (pyridoxine), Active B12, vitamin D, and food intolerancs (IgG) and food allergy (IgE) testing. Participants were asked to withdraw from omega-3 (fish and seafood) and iodine containing supplements for 48 h.

### 2.4 Commercial laboratory analysis

B Vitamins were analyzed via enzyme activation tests at Biolab and Liquid Chromatography with Mass Spectrometry (LC-MS) for Viva Health referrals. Vitamin D was analyzed by either LC-MS at Biolab, or immunoassay at VHL. Iron levels were assessed using spectrophotometric (autoanalyzer method). Iodine levels were analyzed via inductively couple plasma, mass spectrometry (Inductively Coupled Plasma Mass Spectrometry: ICP-MS). Vitamin E was assessed using high performance liquid chromatography at Biolab, or via LC-MS for VHL referrals. Red Cell Magnesium was analyzed from blood samples using atomic absorption spectrometry (AAS) and finally, zinc analyzed using ICP-MS.

#### 2.4.1 Statistical analyses

Statistical analysis was conducted using IBM SPSS Statistics Version 29.0.1.0 (171) and the data split according to laboratory (Viva Health or BioLab). Tests of normality as determined by the Kolmogorov-Smirnov test were applied to the nutritional and psychological data. From the nutritional indices only beta-carotene, vitamin D, iron, zinc and RBC magnesium were normally distributed (*p* < 0.05). Each subscale of the CPRS questionnaire measuring ADHD symptoms, only the *learning language disorder t*-score was normally distributed. Therefore, Pearson coefficients for parametric data and Spearman *rho* coefficients for non-parametric data were conducted. Nutrient levels were assessed to determine whether or not scores met, were above or below the recommended reference ranges. Spearman’s *rho* was applied to explore relationships between individual vitamin, omega–3 PUFA and mineral values and each of the five CPRS DSM-IV *t-*scores e.g., (1) Disruptive Behavior Disorder, (2) Learning & Language, (3) Mood Disorder, (4) Anxiety Disorder and (5) ADHD Index scores. Pearson correlations were employed to assess the relationship between the CPRS Learning & Language Disorder *t-*score and individual nutritional data values (e.g., beta carotene, vitamin D, iron, zinc and RBC magnesium). The *P*-value was considered statistically significant if it was less than 0.05. Corrections for multiple comparisons were not performed on the data given that the primary hypothesis was directional, and data collected in the context of a pilot study.

## 3 Results

### 3.1 Psychological data collection

Forty-six percent of participants had a pre-existing clinical diagnosis of ADHD. Participants either had a pre-existing clinical diagnosis of ADHD or were screened to establish if they met DSM-IV research criteria for ADHD. Screening for children included the completion of (i) Conner Parent Rating Scales (CPRS) (61); (ii) The Children’s Interview for Psychiatric Syndromes (ChIPS) based on DSM-IV criteria (62); (iii), The Kaufman Brief Intelligence tests (K-BIT-2) (63).

### 3.2 Psychological screening

The adult ADHD screening criteria consisted of (i) The Conners’ Adult ADHD Diagnostic Interview for DSM-IV (CAADID) (64); (ii) Adult ADHD Self-Report Scale-V1.1 (ASRS-V1.1) Symptoms Checklist from the World Health Organization (WHO) Composite International Diagnostic Interview (65) and (iii) Depression Anxiety and Stress scales (DASS) (66).

A developmental and medical history was taken and included: (i) diagnostic history and symptoms, (ii) medication; (iii) supplements; (iv) sleep disturbances and average number of hours of sleep; (v) birth weight; (vi) any pregnancy/delivery complications; (vii) antibiotic use.

#### 3.2.1 Participant demographic summary

A total of 35 male and 22 female participants (aged between 4 and 46 years, mean age *M* = 13.5 years, *SD* = 9.6) were recruited for the study. Of which 46% had a pre-existing diagnosis of ADHD, and 19% met screening criteria for the combined subtype of ADHD as measured by The Children’s Interview for Psychiatric Syndromes (ChIPS/P-ChIPS). Sixteen percent of children met “research” criteria for the Predominantly Inattentive Type (ADD). Seventy-two percent of children with ADHD also presented with at least one other comorbidity (e.g., Generalized Anxiety Disorder (GAD) (28%), social communication difficulties (21%), sensory issues (18%), and ASD-related traits/symptoms, 16%).

#### 3.2.2 Medication and/or supplements

Approximately 30% of the study group were taking ADHD medication of which 82% were taking methylphenidate (MPH, e.g., Concerta, Ritalin, Focalin). Fifty-six percent (56%) of the cohort were taking at least one supplement. Popular supplements included omega-3 fatty acids (33%), multivitamin (16%), vitamin D, magnesium (16%), probiotics, vitamin C, melatonin. Out of 44 of the 57 participants, 17 participants (39%) were reported to be *fussy, restrictive, or avoidant* eaters.

#### 3.2.3 Food intolerance testing (IgG)

Thirty-eight participants undertook food intolerance testing. The data revealed that approximately 84% presented with food intolerances to cows’ milk, and 87% to the category “other dairy”. In addition, 53% of the sample presented with food intolerances to wheat; 42% to gluten; 60% to the category “other cereals and seeds”; 29% to yeast and 42% to candida.

#### 3.2.4 Demographic and medical history data

Data was available for 49 of the study group of which approximately 47% of participants experienced complications during delivery (e.g., umbilical cord complications, premature birth) and 33% of participants were delivered via c-section. Approximately, 30% experienced greater than 3 childhood infections and 41% of the children reported (*n* = 49) had been prescribed between 1 and 5 courses of antibiotic medication and 22% had taken 5 or more courses. Intelligent Quotient (IQ) scores were obtained for 42% of the sample *(n* = 24). The mean score for verbal IQ was *M* = 111.7, for non-verbal IQ was 106.7 and for composite IQ was 111.2.

**FIGURE 1:**
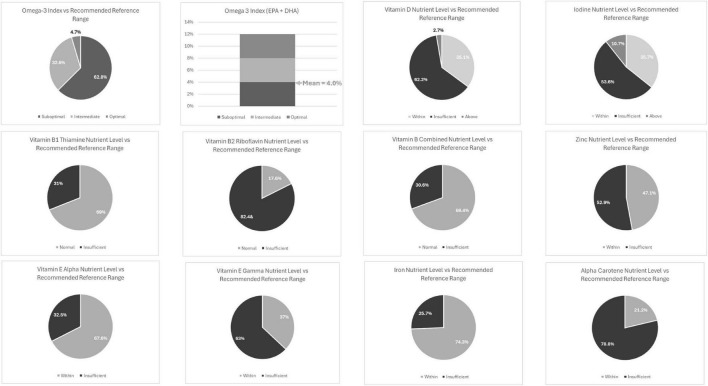
Full cohort nutrient level (%) vs. recommended reference range.

Several nutritional insufficiencies were observed within the total sample including both adults and children (see Table 3) with levels below recommended reference ranges for the following nutrients: vitamin B2, riboflavin (82%), alpha-carotene (79%), vitamin E gamma-tocopherol (63%), vitamin D (62%), iodine (54%), and zinc (53%) (see Figure 1).

**TABLE 3:** Reference ranges for all nutrients according to laboratory (Viva Health and Biolab Medical Unit).

Nutrient	Sample	M and SDs	Reference ranges according to viva health	Sample size	M and SDs	Reference ranges according to BioLab
Vitamin A	6	**1.9 ± 0.9**	2.20 – 4. 00 μmol/L	25	1.6 ± 0.4	1.05 – 2.80 μmol/L
Vitamin E alpha tocopherol	6	**29.2 ± 6.0**	29.5 – 87.4 μmol/L	25	25.7 ± 9.7	25 – 60 μmol/L
Vitamin E gamma tocopherol	6			25	3.3 ± 7.0	2.0 – 8.5 μmol/L
Alpha carotene	6	**0.4 ± 0.4**	0.13 – 0.86 μmol/L	24	1.3 ± 5.7	0.30 – 1.50 μmol/L
Beta carotene	6	0.5 ± 0.4	0.60 – 2.60μmol/L	25	0.8 ± 0.3	0.40 – 3.0 μmol/L
Vitamin B1 thiamine	6	**101.8 ± 74.6**	118 – 235 nmol/L	25	1.1 ± 0.2	< 1.15 = normal
						1.15 – 1.25 borderline
						> 1.30 deficient
Vitamin B2 riboflavin	6	920.5 ± 159.7	797 - 1860 nmol/L	25	**1.4 ± 0.2**	< 1.20 = normal
						1.20 – 1.30 borderline
						> 1.30 deficient
Vitamin B6 pyridoxine	6	105.67 ± 77.1	85-505 nmol/L	25	1.4 ± 0.2	< 1.75 = normal
						1.75 – 2.00 borderline
						> 2.00 deficient
Active B12	5	102 ± 48.2	37.5-150 pmol/L	26	138.9 ± 71.9	25.1 – 165.0 pmol/L
Folate	4	463 ± 346.7	285.4 – 1474.7 nmol/L	25	495.1 ± 265.9	285.4 – 1474.7nmol/L
Vitamin D	6	**50.6 ± 19.1**	82 – 217 nmol/L	25	75.7 ± 16	75 – 200 nmol/L
Iron	6	18.6 ± 4.8	5.83 -34.5 μmol/L	25	16.7 ± 6.4	14.3 – 38.0 μmol/L
Copper	6	15.34 ± 2.2	11.1 – 27.4 μmol/L	25	15.6 ± 3.9	12.5 – 25.0 μmol/L
Magnesium	4	0.9 ± 0.1	0.7 – 1.0 mmol/L	25	0.8 ± 0.2	0.70 – 1.00 mmol/L
Zinc	6	11.7 ± 1.9	10.1 – 20.2 μmol/L	25	**10.8 ± 1.4**	11.5 – 20.0 μmol/L
Red blood cell magnesium	6	2.1 ± 0.2	1.7 – 2.6 mmol/L	24	2.3 ± 0.2	2.08 – 3.00 mmol/L
Iodine (urine)	3	0.6 ± 0.4	0.05-0.36 μmol/L	23	**84.3 ± 89.4**	100 – 199 μg/L

Bold indicates lower than reference range.

#### 3.2.5 Omega-3 Index

Approximately 63% of the full sample presented with *suboptimal* (0–4%) Omega-3 Index, 32.6% had intermediate scores (4–8%) and 4.7% fell in the optimal category (8–12%). The mean Omega-3 Index was *M* = 4.0 SD ± 2.42.

#### 3.2.6 Demographics for subgroup analysis

A subgroup analysis was conducted with a smaller sample (*n* = 36) of children only, comprising 25 males and 11 females (aged between 4 and 18 years, *M* = 9.7 years, *SD* = 3.7) (see Tables 4, 5). All of the sample had a neurodivergent condition of some kind, approximately 86% had a diagnosis of ADHD. Sixty-nine percent of the children within the total sample had at least one other additional behavioral or learning condition, ranging from Speech Language Difficulties (SLD) (25%), GAD (19%), and Dyslexia (14%), respectively. Around a third (32%) of the children were taking ADHD medication, with the majority (82%) prescribed a form of MPH. Around 58% of the children were taking at least one daily supplement (e.g., omega-3, multivitamin, vitamin D, magnesium, probiotics, vitamin C, and melatonin). For example, omega-3 PUFA (22%), multivitamin (19%), magnesium (14%) and melatonin (14%). Participants were asked to refrain from all supplements and stimulant medication for a period of 48-h prior to their blood draw. In relation to eating behaviors, data was available for 29 children, of which 41% were reported to be fussy, restrictive, or avoidant eaters. Food intolerance data was available for 20 children only, of which 80% were found to be intolerant to cows’ milk, and 50% intolerant to wheat. Other food intolerances were found, with 100% of the group (*n* = 20) intolerant to at least one food item.

**TABLE 4:** Demographics and characteristics of the children’s group (*n* = 36).

Demographics: diagnoses & symptoms for the children’s group	Count/*M*+*SD*s	Percentage (%)
**Gender**		
Male	25	69%
Female	11	31%
Age mean (SD)	9.7 ± 3.7	
**Type of ADHD (ChIPS)**		
Predominantly inattentive	7	19%
Predominantly hyperactive/impulsive type	0	0%
Combined type	8	22%
**Pre-existing ADHD diagnosis**		
Predominantly inattentive	4	11%
Predominantly hyperactive/impulsive type	0	0%
Combined type	12	33%
**Any ADHD**	31	86%
**Other diagnosis or symptoms**		
Any other diagnosis	25	69%
Autism spectrum disorder (ASD)	2	6%
ASD traits	5	14%
Dyspraxia	3	8%
Dyslexia	5	14%
Dyscalcula	0	6%
Dysgraphia	2	6%
Obsessive compulsive disorder (OCD) symptoms	1	3%
Epilepsy	1	3%
Depression	1	3%
General anxiety disorder (GAD)	7	19%
Eating disorders	1	3%
Self-harm	2	6%
Hypermobility	3	8%
Tourette’s	4	11%
Oppositional defiant disorder (ODD) or parent-reported aggression	2	6%
Trichotillomania	0	0%
Suicide ideation	0	0%
School phobia	2	6%
Speech language difficulties	9	25%
Sensory or auditory processing disorder	7	3%
Global developmental delay	1	3%
Social communication difficulties	4	11%

**TABLE 5:** Demographic data including medication, supplements, eating styles, food intolerances, childhood infections, antibiotic use and IQ in children and adults with ADHD and other neurodivergent conditions/symptoms in the children’s group (*n* = 36).

ADHD medication	Count/*M+*SDs	Percentage (%)
Methylphenidate (Concerta, Ritalin, Focalin)	9	25%
Amphetamines (Adderall, Vyvanse, Dexedrine)	1	3%
Non stimulants (Atomoxetine, Strattera, Clonidine)	1	3%
**Supplement intake**		
Any supplement	21	58%
Omega-3	8	22%
Multi-vitamin	7	19%
Vitamin D	3	8%
Magnesium	5	14%
Probiotics	1	3%
Vitamin C	2	6%
Melatonin	5	14%
Other supplement use non-specific	5	14%
**Fussy eater, parent-reported (*n* = 29)**		
Yes	12	41%
No	17	59%
**Food (IgG) intolerances (*n* = 20)**		
Any intolerance	20	100%
Cow’s milk	16	80%
Egg yolk	8	40%
Egg white	9	45%
Casein	14	70%
Other dairy	16	80%
Wheat	10	50%
Gluten	5	25%
Other cereals and seeds	11	55%
Yeast	5	25%
Candida albicans	5	25%
Nuts	8	40%
Meats	4	20%
Fish	2	10%
Fruits	6	30%
Vegetables	5	25%
**Complications at birth (*n* = 35)**		
C-section	13	37%
Premature	0	
Umbilical cord around neck	3	9%
**Childhood infections (*n* = 31)**		
Less than 3	20	65%
3 to 5	7	23%
5 or more	1	3%
**Antibiotic courses (*n* = 34)**		
1 to 5	14	41%
5 or more	6	18%
**IQ (Kaufman Brief Intelligence Test, Second Edition (KBIT-2)**		
Verbal	110.5±3.9	
Nonverbal	109.3±7.2	
Composite	114.5±3.7	
**Other IQ test scores**		
Verbal	108.2±10.9	
Nonverbal	101.3±9.8	
Composite	100±10.7	
**Connors’ Parent Rating Scale (CPRS) *t* scores**		
Disruptive behaviour	75.5±17.0	
Learning language disorder	65.9±14.5	
Mood disorder	82.9±10.5	
Anxiety disorder	79.3±11.0	
ADHD indicator	74.2±19.5	

### 3.3 Mode of delivery, antibiotics and IQ

In the children’s group, 50% experienced complications during birth (*n* = 35), 37% were delivered via c-section. Approximately, 22% (*n* = 31) had experienced greater than 3 infections during childhood. Around 41% of children (*n* = 31) had been prescribed between 1 and 5 courses of antibiotic medication and 18% of children had been prescribed 5 or more antibiotic courses.

Children’s IQ scores were assessed for 58% of the sample (*n* = 21). Verbal IQ (*M* = 109.8), non-verbal IQ (*M* = 107.00) and composite IQ (*M* = 110.9).

### 3.4 Children’s results

#### 3.4.1 Nutrient levels

**FIGURE 2:**
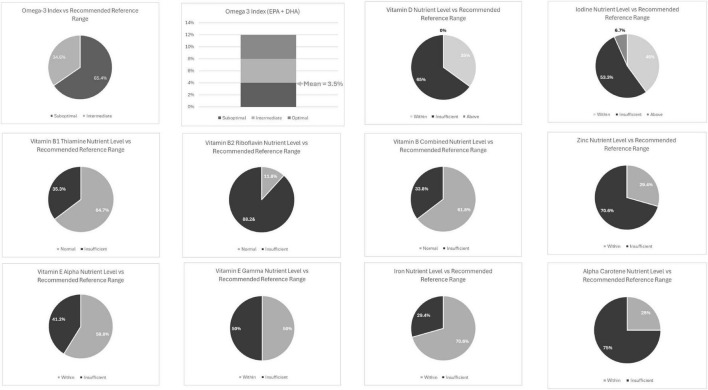
Children’s cohort nutrient levels (%) vs. Recommended Reference Range.

Children presented with several nutrient values below recommended ranges (see Table 6 and Figure 2): vitamin D (65%), iodine (53%), zinc (71%), vitamin B2 Riboflavin (88%), vitamin E gamma-tocopherol (50%) and alpha-carotene (75%) were all below recommended ranges.

**TABLE 6:** Reference ranges for the children’s cohort for all nutrients according to laboratory (viva health and BioLab medical unit).

Nutrient	Sample	M and SDs	Reference ranges according to viva health	Sample size	M and SDs	Reference ranges according to BioLab
Vitamin A	2	**1.94 ± 0.61**	2.20 – 4. 00 μmol/L	15	1.46 ± 0.1	1.05 – 2.80 μmol/L
Vitamin E alpha tocopherol	2	**28.65 ± 0.45**	29.5 – 87.4 μmol/L	15	27.0 ± 3.1	25 – 60 μmol/L
Vitamin E gamma tocopherol	2			15	4.8 ± 2.5	2.0 – 8.5 μmol/L
Alpha carotene	2	0.27 ± 0.18	0.13 – 0.86 μmol/L	15	0.2 ± 0.02	0.30 – 1.50 μmol/L
Beta carotene	2	**0.12 ± 0.07**	0.60 – 2.60μmol/L	15	1.0 ± 0.1	0.40 – 3.0 μmol/L
Vitamin B1 thiamine	2	**28.5 ± 0.5**	118 – 235 nmol/L	15	1.5 ± 0.02	< 1.15 = normal 1.15 – 1.25 borderline > 1.30 deficient
Vitamin B2 riboflavin	2	819.5 ± 84.5	797 - 1860 nmol/L	15	**1.4 ± 0.05**	< 1.20 = normal 1.20 – 1.30 borderline > 1.30 deficient
Vitamin B6 pyridoxine	2	**78.5 ± 26.50**	85-505 nmol/L	15	1.4 ± 0.06	< 1.75 = normal 1.75 – 2.00 borderline > 2.00 deficient
Active B12	2	73.2 ± 27.8	37.5-150 pmol/L	15	136.8 ± 17.5	25.1 – 165.0 pmol/L
Folate	2	258.0 ± 28.0	285.4 – 1474.7 nmol/L	15	497.9 ± 43.3	285.4 – 1474.7nmol/L
Vitamin D	2	**47.05 ± 13.25**	82 – 217 nmol/L	15	**74.5 ± 4.1**	75 – 200 nmol/L
Iron	2	20.9 ± 4.2	5.83 -34.5 μmol/L	15	15.2 ± 1.6	14.3 – 38.0 μmol/L
Copper	2	13.7 ± 1.2	11.1 – 27.4 μmol/L	15	17.1 ± 0.8	12.5 – 25.0 μmol/L
Magnesium	2		0.7 – 1.0 mmol/L	15	0.86 ± 0.01	0.70 – 1.00 mmol/L
Zinc	2	10.7 ± 0.6	10.1 – 20.2 μmol/L	15	**10.8 ± 0.4**	11.5 – 20.0 μmol/L
Red blood cell magnesium	2	2.0 ± 0.1	1.7 – 2.6 mmol/L	15	2.3 ± 0.1	2.08 – 3.00 mmol/L
Iodine (urine)	2	0.63	0.05-0.36 μmol/L	15	**86.7 ± 21.1**	100 – 199 μg/L

Bold indicates lower than reference range.

#### 3.4.2 Omega-3 Index

65% of the children presented with suboptimal Omega-3 Index scores. Intermediate Omega-3 Index values were found in 27% (intermediate = 4–8%). None of the children had optimal Omega-3 Index values (optimal = 8–12%). The mean score for the cohort was *M* = 4.35 SD ± 1.98.

#### 3.4.3 ADHD symptoms and nutrient levels

The following associations between nutrient values and ADHD symptoms scores were observed.

#### 3.4.4 CPRS disruptive behavior index and RBC magnesium

A statistically significant, moderate, negative correlation was observed between RBC magnesium and the CPRS Disruptive Behavior subscale scores, (*rho* = −0.597), *p* = 0.024.

#### 3.4.5 CPRS Learning Language Disorder index and omega-3 index scores

A moderate, negative statistically significant correlation was observed between omega-3 index scores and the CPRS Learning Language Disorder subscale scores, (*rho* = -0.601, *p* = 0.018).

#### 3.4.6 Vitamin B2 and CPRS learning and language disorder index

A positive (but not significant) relationship was observed between Vitamin B2 (deficient variable) and the CPRS Learning and Language Disorder subscale scores (*rho* = 0.489, *p* = 0.076).

#### 3.4.7 Beta carotene and CPRS mood disorder index

A moderate negative, non-significant correlation was observed between beta carotene values and the CPRS Mood Disorder subscale scores, (*rho* = -0.497, *p* = 0.071).

#### 3.4.8 Vitamin B1 (thiamine) and CPRS anxiety disorder index

A moderate positive, statistically significant correlation was observed between vitamin B1 value and CPRS Anxiety Disorder subscale scores, (*rho* = 0.584, *p* = 0.028).

#### 3.4.9 Alpha-carotene and CPRS ADHD indicator index

A moderate negative, statistically significant correlation was observed between alpha-carotene values and total ADHD Index scores (*rho* = −0.617, *p* = 0.012).

#### 3.4.10 RBC Magnesium and CPRS ADHD index

A moderate negative, statistically significant correlation was observed between RBC magnesium and total ADHD index scores (*rho* = −0.612, *p* = 0.02). No further relationships were observed.

## 4 Discussion

The relationship between nutrition and ADHD is gaining increasing scientific and public attention, although many unanswered questions remain. This study sought to provide insights into some of these complex patterns and reveal some preliminary observations which can provide a catalyst for further research. The participants personalized nutrient profiles were measured using blood samples and the results compared to recommended daily intakes (RDI). An RDI is a standardized guide used in the UK and EU to indicate the average amount of nutrients an individual should consume daily. The main findings of this study are that children and adults with ADHD and other neurodivergent conditions and symptoms presented with a range of nutrient levels below recommended reference ranges. The majority of neurodivergent adults (95%) presented with suboptimal omega-3 index scores and none of the children had optimal omega-3 fatty acid levels. Children and adults with ADHD and other neurodiverse conditions had at least one food intolerance and the majority presented with food intolerances to cow’s milk, other diary, and to a lesser extent wheat and gluten. Several significant relationships were observed between ADHD symptoms and nutrient levels providing interesting insights albeit in a small sample size. This study provides information regarding intakes of key nutrients linked to healthy neurotransmitter function which in turn are implicated in ADHD and associated brain (/mental) health symptoms.

### 4.1 Omega-3 fatty acids

There is robust evidence of the importance of omega-3 PUFAs for brain development and throughout the lifespan (67–69). Omega-3 PUFA insufficiencies are related to ADHD, autism, dyslexia, depression, sleep disturbances, dysregulated mood and attention deficits (4, 6, 24, 70–75). The finding of lower blood levels of omega-3 fatty acids have been consistently reported in ADHD and depression (49, 50, 73, 76). Conversely, supplementation with omega–3 PUFA has been found in several studies to improve attention-deficits, literacy and cognitive outcomes (77, 78) as well as callous and unemotional traits (CU) and antisocial behaviors (74, 75). Increasing evidence suggests that omega–3 PUFAs in combination with other nutrients may have a protective and inhibitory role and is implicated in a range of brain health and visual conditions (79, 80). Emerging research findings position nutrition as an epigenetic neuromodulator with the ability to modify gene expression at transcriptional levels (81–84).

The results of this study demonstrated that 95% of participants in the full cohort presented with a suboptimal omega-3 index and furthermore the omega-3 index was negatively associated with learning and language disorder scores. Several other published studies support the finding of a low omega-3 index scores. First, the DOLAB study, reported a mean omega-3 index score of 4.23% in UK school children (85). Meanwhile, Parletta et al. (37) reported a mean omega–3 index score of 3.95% in individuals with schizophrenia and depression (37). The NORAA trial (Neuroimaging, Omega-3 and Reward in Adults with ADHD clinical trial, unpublished data) observed an omega-3 index of 4.33% in adults with ADHD (*n* = 36) (86, 87). Additionally, a recent study reported an omega-3 index of 5.5% in young adults with subthreshold depression (88). Collectively, these studies support our finding of low omega-3 index scores in clinical populations. The finding of low omega-3 fatty acids may directly reflect a lack of dietary intake from fish/seafood. However, a third of participants (in the full cohort) reported taking fish oil supplements although use was inconsistent, and the type and form were not recorded. There is the possibility of potential problematic absorption and impaired synthesis as reported by others but that is beyond the scope of this study (89, 90). Omega-6 and omega-3 PUFAs compete for absorption and a diet high in omega–6 PUFAs decreases the levels omega-3 PUFAs (91). Almost half of the children (41%) in this study were reported as fussy eaters.

### 4.2 B-vitamins

This study revealed that the majority of participants (in the full cohort) had insufficient vitamin B2 levels (riboflavin) and furthermore, that low vitamin B2 was negatively associated with higher learning and language disorder scores. Previous studies have reported that lower levels of vitamin B2 are associated with greater severity of ADHD symptoms (51). B-vitamins are critical for neurotransmitter function and in particular help regulate gamma-aminobutyric acid (GABA), dopamine, serotonin and norepinephrine (92). Dopamine is critical for motivation and reward-related processes and dopamine deficits are considered a hallmark feature of ADHD (93). GABA helps reduce hyperactivity, anxiety and is critical for sleep regulation (94). Lower levels of GABA are linked to depression, stress and anxiety (95, 96) and shorter sleep duration (97). Vitamin B6 assists in the production of serotonin, and supplementation with B6 has resulted in self-reported reductions in anxiety (98). Several research studies have reported that high-dose vitamin B supplementation may be effective in reducing symptoms of anxiety (98, 99). In addition to assisting in the production of serotonin and melatonin, B-vitamins also help produce norepinephrine and lower levels can lead to an overactive noradrenergic system resulting in social and behavioral problems (100). This study revealed lower vitamin B2 in children and adults with ADHD and other neurodivergent associated conditions. Landass and colleagues recently examined the vitamin status of 131 young adults with ADHD compared to controls and reported lower vitamins B2, B6 and B9 were related to their ADHD diagnosis, and vitamins B2 and B6 with symptom severity (51).

### 4.3 Magnesium

This study reported that lower levels of magnesium were negatively associated with both disruptive behavior and ADHD symptom scores. This finding supports research by Portnoy et al. (101) who reported that lower dietary intake of magnesium is associated with higher scores of callous and unemotional traits (CU) in children (101). Over a decade of research has presented findings of lower levels of magnesium in children and adults with ADHD (102–105). Conversely, higher dietary intake of magnesium is associated with improved emotional, conduct, social and peer problems in children with ADHD (106–108).

### 4.4 Zinc

This study’s findings observed that over half of the full cohort presented with levels of zinc below recommended reference ranges. Insufficient levels of zinc have been linked to mood instability, antisocial behavior and learning problems (109–111). A small research study in 58 children with ADHD reported significantly lower zinc, ferritin and magnesium status compared to controls (112). Collectively, zinc status in ADHD has yielded mixed findings and due to the complexity and heterogeneity of nutrition research this is not unusual (113, 114). Zinc is an important cofactor directly impacting dopamine metabolism and also relevant to prostaglandin and melatonin activity (109). As a cofactor for enzymes involved in dopamine synthesis and transport, zinc insufficiency may contribute to ADHD symptoms through dysregulated dopamine pathways (115). Low zinc may also interfere with psychostimulant medications for ADHD with higher zinc enabling a lower dose of MPH (109). It may also play a preventative role in the development of mood disorders and has been demonstrated to improve the efficacy of antidepressants in treatment-resistant patients (116). The copper-zinc ratio and balance is relevant given that excess copper (Cu) depletes zinc (Zn) in the body and increases risk of insufficiency/deficiency. The Cu:Zn ratio has been found to be significantly higher in children with ADHD than controls and may significantly contribute to ADHD variability (117).

### 4.5 Antioxidants

The antioxidants alpha-carotene and vitamin E were lower than recommended reference ranges in 80 and 63% of the full cohort, respectively. Reduced antioxidant enzyme activity and specifically vitamin E has been reported in children with ADHD (118). Additionally, oxidative stress and inflammation are both influenced by the diet and implicated in ADHD and other metabolic health conditions (119–122). Omega-3s contain antioxidant and anti-inflammatory properties and supplementation has resulted in small-modest effect sizes in reducing clinical symptoms of ADHD (53). In addition, zinc influences antioxidant defense mechanisms, and fat-soluble vitamins D and E regulate the production of immune cells and help combat inflammation (123). Given the children and adults with ADHD in this study also presented with low omega–3 and to a lesser extent low zinc, these collective findings may have important implications for future exploration in oxidative stress pathways in ADHD (119, 121). There is little research on alpha-carotene specifically in ADHD, however, it is converted to retinol (Vitamin A) in the body and supports immune system functioning. The MIND study reported that higher circulating alpha-carotene was associated with improved cognitive function in adults at risk for cognitive decline (124). Lower vitamin A and vitamin D have been observed in children with ADHD and were linked to a worsening of symptoms (125). Both vitamin A and vitamin D work synergistically to regulate gene expression and support the immune system (126). This study also reported a negative correlation between alpha-carotene and ADHD symptoms, indicating that lower alpha-carotene is related to greater ADHD symptom severity.

### 4.6 Vitamin D

Around sixty-two percent of children and adults with ADHD presented with low levels of vitamin D. A systematic review and meta-analysis examined vitamin D status in 10,334 children and adolescents with ADHD compared to controls (127). A small study by Sharif et al. (128) reported significantly lower levels of serum vitamin D in children with ADHD (*n* = 37) compared to controls (*n* = 37) (128). Another systematic review and meta-analysis reported that vitamin D had a moderately favorable antidepressant effect in lowering symptoms of depression and anxiety (129). Vitamin D and omega-3 support the production of serotonin which has specific relevance for ADHD and mood. Patrick and Ames (130) report that vitamin D insufficiencies (found in approximately 70% of the population) and inadequate omega-3 fatty acids levels are common and that this is likely to impact serotonin levels (130). A systematic review and meta-analysis examined the adjunct effects of vitamin D in 256 children taking MPH and found a small, but statistically significant benefit in reducing symptoms (131). Goksugur et al. (132) reported significantly lower serum vitamin D in children and adolescents with ADHD compared to controls (132). ADHD is commonly accompanied by low mood, depression and anxiety, and therefore optimal levels of vitamin D should be carefully monitored (133).

### 4.7 Iodine

Over half of children and adults with ADHD and neurodivergence presented with low levels of iodine. There were no relationships with ADHD symptom severity. Iodine is an essential trace element needed in the diet. Iodine insufficiency is linked to a range of complications including cognition, lower IQ and risk of thyroid disorders in adults (134). A link between insufficient iodine levels during pregnancy and increased risk of ADHD symptoms has been reported (135). The cognitive effects of iodine insufficiency in children with ADHD has been explored and relationships between low iodine and higher incidence of learning problems with consequences for neurodevelopmental conditions (136, 137).

### 4.8 Food intolerances (IgG)

Research dating back to almost 100 years ago identified increased restlessness and sleep disturbances in children with food intolerances (138). Following the removal of specific food items, they reported a reduction or disappearance of symptoms (138). In the twenty-first century, modern research continues to acknowledge the benefits of a restriction diet in some children with ADHD (139). The growing inclusion of food additives, i.e., flavoring, preservatives and dyes in ultraprocessed foods is of current global concern (18). UPFs are linked to problematic behavior and poor gut health and arguably children are the most vulnerable to potential harms (140–143). Over a decade ago, McCann et al. (144) reported that sodium benzoate in juice versus a placebo drink resulted in increased hyperactivity in children (144). Children with ADHD are commonly found to present with a variety of food sensitivities and intolerances including sugar, dairy, wheat and wheat gluten (145). This study reports a range of high IgG reactivity scores to dairy, wheat, and gluten in children and adults with ADHD. Almost 85% of the cohort presented with a cow’s milk intolerance. There is a history of cow’s milk intolerance associated with ADHD and in fact around 65% of the population are considered to be intolerant. The milk protein casein is converted to casomorphin (morphine-type compounds) in the body which attach to opiate receptors in the brain (146). Elevated amounts of casoporphin are often linked to inactive “dipeptidyl peptidase IV” (DPP-IV) enzyme activity and may lead to symptoms of brain fog, irritability, aggression, anxiety and depression, fatigue, sleep and mood problems (147, 148). Gluten is also converted into an opioid peptide called gliadomorphin and gluteomorphin and effects align with casomorphins. Both are hypothesized to impact gut health and are implicated in a range of inflammatory and autoimmune conditions (146). Emerging discoveries and research into the gut-brain axis casts no doubt that this intricate relationship has a wide-range of bodily and brain health impacts influencing neurodevelopment and behavior (149–152). Food cravings are likely to be indicative of the balance and diversity of gut microbiome, highlighting a connection between food preferences and microbiome composition (153). From this perspective, children with ADHD and (undiagnosed) food intolerances may crave the very foods they are in fact intolerant to reflecting a type of microbial manipulation (154–156). The link between gut health and ADHD and neurodivergent conditions warrant further exploration (157–159).

It is important to note that conducting nutritional research is challenging often because isolating a specific nutrient and then supplementing is too simplistic and arguably outdated. There is a growing need to better understand the concept of nutritional synergy by researchers to optmise clinical trial outcomes (2). Within ADHD and omega-3 research specifically, Bloch et al. (160) has previously highlighted repeated issues in published nutritional studies, including missing power calculations, small sample sizes, and inconsistencies in design, methodology, micronutrient dosage, duration, and baseline measurements (161). A holistic, personalized and integrative approach in nutritional research is recommended. For example, involving a combination of a range of assessments including nutrient profiles from blood draws, measures of gut health, e.g., stool samples, food records of daily nutrient intake and food intolerance/allergy testing to permit a clearer, informed, picture to evolve.

### 4.9 Strengths and limitations

This study conducted detailed self-report educational and medical histories, the completion of psychological questionnaires, and nutritional data with families seeking consultancy within private practice. This enhances external validity by reflecting real-world conditions, making findings more applicable to everyday settings. Blood samples are considered a reliable and robust method to measure an individual’s nutritional status and to detect nutritional insufficiencies compared to self-reported food dairies (162). However, the study faced several limitations, including the absence of a comparison group (i.e., non-diagnosed age and sex-matched controls), potentially influencing the interpretation of the results. The study also acknowledges that the smaller sample size may inadequately represent diverse demographics. Additionally, the majority of participants were from high-income families, who could afford to pay for nutritional testing. Variations in genetic and lifestyle factors that influence nutrient metabolism were not explored.

## 5 Conclusion

This study presents for the first time preliminary data about nutritional blood level status in ADHD and other neurodivergent conditions. The nutritional insufficiencies namely, omega-3 PUFAs, zinc, vitamin E, alpha-carotene, vitamin D, vitamin B2, iodine and magnesium, respectively, are linked to neurotransmitter function which arguably have implications for learning, behavior and mood. Furthermore, the study findings identified relationships between some of these key nutrients and ADHD symptoms which may underlie brain activity and neurotransmitter function. Finally, it highlighted several food intolerances which are speculated to relate to food cravings and gut health and warrant further exploration. Investigations in gut health and ADHD and ASD is of current scientific interest, and emerging data has highlighted links between an autism diagnosis and gut health disturbances. Food intolerances and gut dysbiosis are linked, and diet is a critical factor that affects gut microbiome. Our preliminary, observational study highlights the need for larger clinical research investigating nutrient intakes in the wider UK population and a control group. Future research studies should further investigate the role of brain-selective nutrients in children and adults with and without ADHD with larger sample sizes to better understand their collective influence in brain health, learning, behavior and mood.

## Data Availability

The data that support the findings of this study are available from the corresponding author RVG, upon reasonable request.

## Footnotes

1
https://www.nice.org.uk/guidance/ng87
